# Diverse RNA viruses discovered in multiple seagrass species

**DOI:** 10.1371/journal.pone.0302314

**Published:** 2024-08-28

**Authors:** Jordan E. Rede, Mya Breitbart, Carolyn Lundquist, Keizo Nagasaki, Ian Hewson

**Affiliations:** 1 Department of Microbiology, Cornell University, Ithaca, NY, United States of America; 2 College of Marine Science, University of South Florida, Saint Petersburg, FL, United States of America; 3 National Institute of Water and Atmospheric Research, Hamilton, New Zealand; 4 School of Environment, The University of Auckland, Auckland, New Zealand; 5 Faculty of Science and Technology, Kochi University, Nankoku, Kochi, Japan; Nuclear Science and Technology Research Institute, ISLAMIC REPUBLIC OF IRAN

## Abstract

Seagrasses are marine angiosperms that form highly productive and diverse ecosystems. These ecosystems, however, are declining worldwide. Plant-associated microbes affect critical functions like nutrient uptake and pathogen resistance, which has led to an interest in the seagrass microbiome. However, despite their significant role in plant ecology, viruses have only recently garnered attention in seagrass species. In this study, we produced original data and mined publicly available transcriptomes to advance our understanding of RNA viral diversity in *Zostera marina*, *Zostera muelleri*, *Zostera japonica*, and *Cymodocea nodosa*. In *Z*. *marina*, we present evidence for additional Zostera marina amalgavirus 1 and 2 genotypes, and a complete genome for an alphaendornavirus previously evidenced by an RNA-dependent RNA polymerase gene fragment. In *Z*. *muelleri*, we present evidence for a second complete alphaendornavirus and near complete furovirus. Both are novel, and, to the best of our knowledge, this marks the first report of a furovirus infection naturally occurring outside of cereal grasses. In *Z*. *japonica*, we discovered genome fragments that belong to a novel strain of cucumber mosaic virus, a prolific pathogen that depends largely on aphid vectoring for host-to-host transmission. Lastly, in *C*. *nodosa*, we discovered two contigs that belong to a novel virus in the family *Betaflexiviridae*. These findings expand our knowledge of viral diversity in seagrasses and provide insight into seagrass viral ecology.

## Introduction

Seagrasses are an integral part of the marine environment. Their leaves support grazing and herbivory and their meadows form complex ecosystems that enrich biodiversity [1–4]. These qualities derive from their high productivity and structural complexity, but seagrasses also provide a number of functions that are important for ecosystem health. Their roots stabilize sediments and drive nutrient cycling through radial oxygen loss [5, 6], and their canopies improve water quality and help sequester carbon through particle baffling in the water column [7, 8]. Seagrass die-offs therefore have a negative effect on marine ecosystems. While some regions have seen seagrass populations rebound [9, 10], seagrasses are declining worldwide [11, 12]. Seagrass mortality can occur through natural causes like wasting disease [13, 14], but anthropogenic factors (e.g., light limitation from nutrient contamination and algal growth) account for the majority of seagrass die-offs [15, 16]. In light of their importance, numerous studies have sought to characterize the seagrass microbiome [17–19]. Notably, however, the vast majority of these studies have focused on bacteria, which neglects potential impacts from non-cellular constituents like viruses.

Viruses have an underappreciated role in plant ecology. The majority of research relies on a limited number of pathosystems, which biases our understanding of virus-host interactions on a broad scale [20]. In wild plants, viral infections are frequent but often asymptomatic [21], and they can produce a variety of effects that range from deleterious to beneficial, conferring advantages like drought/cold tolerance and pathogen exclusion (i.e., mechanisms that prevent pathogen entry into cells) [22]. Virus research in marine plants is extremely limited, and studying seagrass virology will help us better understand the effect that viruses have on seagrass ecology. Furthermore, comparisons between virus-host dynamics in marine and terrestrial plant systems could advance our understanding of plant viruses generally. Recently, several papers have begun to address the diversity of the seagrass virome [23–28], but the field of seagrass virology is still young with a great deal of diversity undiscovered.

In this study, we aimed to broadly survey RNA viruses in four seagrass species: *Zostera marina*, *Zostera muelleri*, *Zostera japonica*, and *Cymodocea nodosa*. We prepared viral transcriptomes from leaf tissue collected in the United States, Japan, and New Zealand, and surveyed publicly available transcriptomes from NCBI. Including both original and public data, we present new genotypes and novel viruses from the families *Amalgaviridae*, *Endornaviridae*, *Betaflexiviridae*, *Bromoviridae*, and *Virgaviridae*.

## Materials and methods

### Sample collection

*Z*. *marina* and *Z*. *muelleri* leaf tissues were collected from three locations: Kochi, Japan; Massachusetts (MA), United States of America (USA); and Whangārei, New Zealand. All tissues were randomly sampled from ostensibly healthy populations. In Kochi, Japan, leaf tissue from eight *Z*. *marina* plants were collected from the Uranouchi inlet in May 2020. These samples were stored in RNA*later*^TM^ (Invitrogen^TM^, USA) and kept at -20°C before transport to Cornell University, Ithaca, NY, USA. Ten *Z*. *marina* samples from independent plants were also collected from MA, USA, in West Falmouth Harbor. These were collected in July 2021 and flash frozen in liquid N_2_ before transport to Cornell University. A total of 36 *Z*. *muelleri* plants were sampled in Whangārei, New Zealand–six from each of the following field sites: McDonald Bank, Munro Bay, One Tree Point, Takahiwai, Snake Bank, and Taurikura. These were collected in November 2020 and also stored in RNA*later*^TM^ before transport. After arriving at Cornell University, all samples were stored at -80°C until further processing. In addition to our survey, transcriptomes (*C*. *nodosa*–ERR1211085-93 [29], *Z*. *japonica*–SRR8149505-10 [30], and *Z*. *marina*–SRR4241949-50, SRR4241952-53 [31]) were mined from the NCBI Sequence Read Archive (SRA).

### Sample processing and library preparation

Seagrass tissues were initially thawed on ice. Samples stored in RNA*later*^TM^ were also rinsed with 0.02 µm filtered phosphate buffered saline (PBS) and combined for library preparation. The eight samples from Japan were condensed into four libraries, combining tissues from two plants into each library. Likewise, the libraries from New Zealand (n = 6; one per field site) were made using tissue samples from two independent plants. Flash freezing samples allowed us to collect larger leaf sections, negating the need to combine tissues from the USA. For each library, approximately 200 mg of tissue was homogenized in 750µL of 0.02 µm filtered PBS for 1 min using ZR BashingBead^TM^ Lysis Tubes (0.1 and 0.5 mm; Zymo Research, USA) and a Mini BeadBeater-8. Samples were then centrifuged at 5000 x g for 5 min. The resulting supernatant was pushed through a 0.2 µm polyethersulfone (PES) filter and treated with 50U of RNase One^TM^ Ribonuclease (Promega^TM^, USA) and 5U of TURBO™ DNase (Invitrogen^TM^, USA) for two hours at 37ºC. Immediately following incubation, viral RNA was extracted using a *Quick*-RNA^TM^ Viral Kit (Zymo Research, USA) in accordance with the recommended protocol. After RNA extraction, each sample was filtered through a Zymo-Spin^TM^ III-HRC filter (Zymo Research, USA) to remove PCR inhibitors and stored at -80ºC until library preparation. Transcriptome libraries were prepared using a TransPlex^®^ Complete Whole Transcriptome Amplification Kit (Sigma-Aldrich^TM^, USA) in accordance with the recommended protocol. The final PCR products were then cleaned with a Clean and Concentrator^®^-5 kit (Zymo Research, USA) and sequenced (2 x 250 paired-end) using an Illumina MiSeq platform at the Cornell Biotechnology Resource Center, Ithaca, NY. In total, we sequenced four libraries from Kochi, Japan, five libraries from MA, USA, and six libraries from Whangārei, New Zealand. These libraries can be found in the NCBI SRA database under BioProject PRJNA936818.

### Bioinformatics pipeline

Libraries prepared in-house were processed and assembled in groups based on geography. Reads derived from the SRA were processed and assembled in groups according to their BioProject accession. Initial library qualities were first assessed using FastQC (v. 0.11.9) and error corrected using Rcorrector (v. 1.0.4) [32]. Read pairs that were unfixable were discarded. Reads were then quality controlled and trimmed using Trim Galore (v. 0.6.5) and mapped to an rRNA database to remove ribosomal sequences. To construct the rRNA database, the SSU and LSU Parc datasets were downloaded from the SILVA database (132 release) and indexed using Bowtie 2 (v. 2.4.1) [33]. Lastly, the processed reads were assembled into contigs using Trinity (v. 2.11.0) [34]. Assembled contigs were queried against a custom database of plant viral proteins using Diamond BLASTx (v. 2.0.4) [35]. To construct the database, viral protein sequences were downloaded from the NCBI RefSeq database and then indexed using the makeblastdb command in Diamond. These include proteins from the taxonomic groups *Amalgaviridae*, *Bromoviridae*, *Caulimoviridae*, *Closteroviridae*, *Endornaviridae*, *Fimoviridae*, *Geminiviridae*, *Partitiviridae*, *Potyviridae*, *Rhabdoviridae*, *Secoviridae*, *Solemoviridae*, *Tombusviridae*, *Tospoviridae*, *Tymovirales*, and *Virgaviridae*. Positive hits (E-value < 1e-20) were vetted in a holistic manner based on identifiable open reading frames (ORFs), protein families, and sequence length. After viral contigs were identified, their mean depth was calculated using the SAMtools (v. 1.17) coverage command on a Bowtie 2 generated alignment [36]. Each alignment was generated using the same group of samples that were used to assemble the contigs. All downstream analyses were performed using Geneious Prime (v. 2021.2.1) and the R programming language (v. 4.2.0) [37].

### Contamination control

The viral contigs found in tissues collected for this study were cross-checked based on location (Japan, USA, and New Zealand) to rule out the possibility of reagent contamination and cross-contamination between libraries. The ability to control for contamination in publicly available transcriptomes is limited, unfortunately. However, there is an established precedent for data mining in virus discovery that includes seagrass viruses [24, 25, 27, 28]. Therefore, viral contigs with sufficient coverage, identifiable ORFs, and protein coding regions were included.

### Phylogenetics

To construct phylogenies, amino acid or nucleotide sequences were first aligned using the GUIDANCE2 webserver and MAFFT multiple sequence alignment (MSA) algorithm [38, 39]. With the exception of cucumber mosaic virus (CMV), amino acids were used for each alignment. For CMV, nucleotides were used in lieu of amino acids because of the high similarity between CMV strains. Alignments were exported from the GUIDANCE2 webserver and imported into Geneious Prime, where they were trimmed to equal lengths and manually refined. Regions with low confidence GUIDANCE2 scores were removed. Phylogenies were constructed in Geneious Prime using the PHYML plugin with default settings–an LG substitution model with Shimodaira–Hasegawa (SH)-like branch support [40].

### Protein identification and transmembrane discovery

Protein coding regions were identified using the InterProScan plugin in Geneious Prime [41]. Putative transmembrane protein domains were identified using the CCTOP webserver [42].

### Figures

Phylogenies were exported and remade in R (v. 4.2.0) using the following packages–APE (v. 5.6–2), ggtree (v. 3.4.0), and phytools (v. 1.2–0) [43–45]. Circos plots, MSAs, and synteny plots were made using the circlize (v. 0.4.15), ggmsa (v. 1.2.3), and gggenomes (v. 0.9.5.9000) packages, respectively [46–48].

## Results and discussion

We assembled contigs from four seagrass species (*C*. *nodosa*, *Z*. *marina*, *Z*. *muelleri*, and *Z*. *japonica*) that belong to five viral families: the *Amalgaviridae*, *Endornaviridae*, *Betaflexiviridae*, *Bromoviridae*, and *Virgaviridae*. Table 1 summarizes the viruses found in this study in addition to previously discovered seagrass viruses. S1 Table summarizes the mean sequencing depth for viruses presented in this study. Predictably, fragmented genomes with shorter contigs had a lower mean depth compared to complete or near complete genomes.

**Table 1:** A list of known seagrass viruses and their hosts.

Host Species	Virus	Genome	Study	Data	Country	BioProject
*Cymodocea nodosa*	Cymodocea alphaflexivirus 1	+ ssRNA	[27]	PDG	N/K	PRJNA275569
	Cymodocea nodosa foveavirus 1	+ ssRNA	[27]	PDG	N/K	PRJNA275569
	Cymodocea nodosa betaflexivirus	+ ssRNA	TS	PUB	ES	PRJEB12372
*Thalassia testudinum*	Turtlegrass virus X	+ ssRNA	[26]	PDG	US	N/A
*Zostera marina*	Zostera associated varicosavirus 1	- ssRNA	[28]	PDG	CN	PRJNA609020
	Zostera marina alphaendornavirus	+ ssRNA	TS	NEW	JP	PRJNA936818
	Zostera marina alphaendornavirus 1	dsRNA	[24]	PDG	CN	PRJNA235360
	Zostera marina alphaendornavirus 2	dsRNA	[24]	PDG	CN	PRJNA235360
	Zostera marina amalgavirus 1-like SD	dsRNA	TS	PUB	CN	PRJNA342750
	Zostera marina amalgavirus 2-like KG	dsRNA	TS	NEW	JP	PRJNA936818
*Zostera muelleri*	Zostera muelleri associated alphaendornavirus	+ ssRNA	TS	NEW	NZ	PRJNA936818
	Zostera muelleri furovirus	+ ssRNA	TS	NEW	NZ	PRJNA936818
	Zostera virus T	+ ssRNA	[25]	PDG	AU	PRJEB9377
*Zostera japonica*	Cucumber mosaic virus	+ ssRNA	TS	PUB	CN	PRJNA503298

The genome column indicates if the virus genome is positive or negative-sense single-stranded (ss) RNA or double-stranded (ds) RNA. TS in the study column indicates the virus came from this study. A designation of NEW in the data column means the genome came from original data from this study, whereas PUB indicates the genome came from public transcriptomes. PDG indicates a previously discovered genome. For country of origin, ES = Spain, US = United States, JP = Japan, CN = China, and AU = Australia. N/K indicates the country is not known. In the BioProject column, N/A (not applicable) indicates that no BioProject is available.

### Amalgaviridae

We found two amalgavirus genomes in the seagrass *Z*. *marina*. The amalgaviruses are vertically transmitted, persistent viruses with no discernible pathology [49]. They have dsRNA genomes, a single known protein, RNA-dependent RNA Polymerase (RdRP), and may be capsidless [50–52]. Two amalgavirus genomes have been discovered in seagrass previously, Zostera marina amalgavirus 1 (ZmAV1) and Zostera marina amalgavirus 2 (ZmAV2) [24]. The first genome we found is near-complete and most similar to ZmAV1, and it was assembled from transcriptomes originally published by Lv *et al*. [31]. The second genome we found is partially complete and most similar to ZmAV2, and it was detected in sequence libraries prepared from Japan for this study. Here, we differentiate between genotypes by using the designations ZmAV1-like Shuangdao genotype (ZmAV1-SD; China) and ZmAV2-like Kochi genotype (ZmAV2-KG; Japan), both named for their geographic origin.

**Fig 1:**
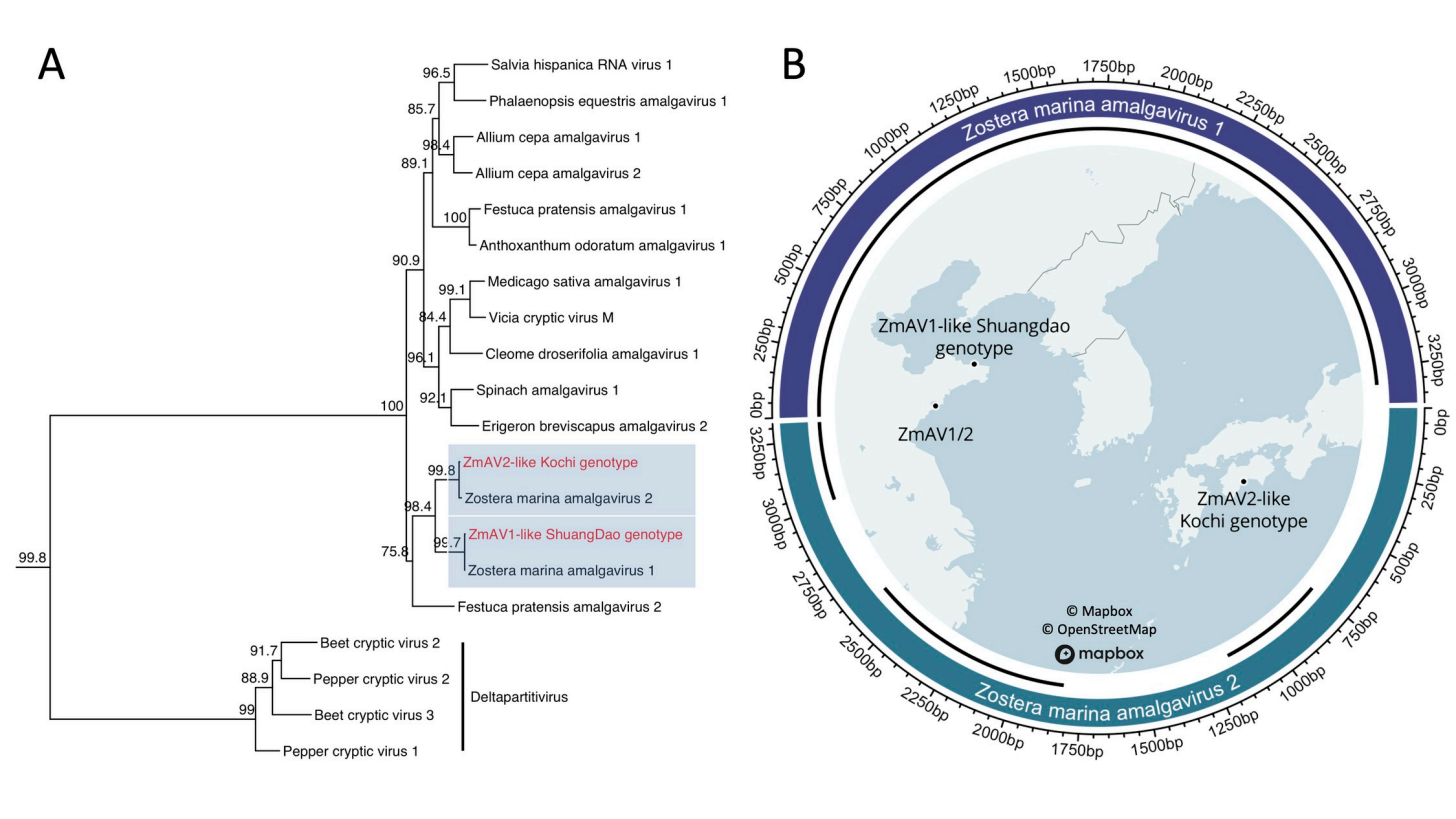
Phylogeny and contig alignments for new amalgavirus genomes. (A) Phylogenetic placement of *Z*. *marina* amalgaviruses using a maximum-likelihood inference. This phylogeny was constructed with an amino acid alignment of amalgavirus and deltapartitivirus RdRP domains. Numeric values indicate the degree of SH-like branch support (scale 0–100). (B) Circlize plot of ZmAV1-like Shuangdao genotype (ZmAV1-SD) and ZmAV2-like Kochi genotype (ZmAV2-KG) contigs mapped to Zostera marina amalgavirus 1 and Zostera marina amalgavirus 2 (ZmAV1/2) genomes. Track 1 represents the complete length of ZmAV1 (top) and ZmAV2 (bottom). Scale markers on the outside of track 1 are in base pairs. Track 2 represents ZmAV1-SD and ZmAV2-KG contigs and their genomic position relative to ZmAV1 and ZmAV2. Internal map shows the field site where all *Z*. *marina* amalgaviruses were found, and was printed under a CC BY license with permission from Mapbox.

The ZmAV1-SD assembly produced a single 3,313 nt long contig representing a near complete genome (Fig 1B). This contig has two overlapping ORFs and a +1 ribosomal frameshift motif, consistent with other amalgaviruses [53]. Its RdRP region is 99.29% similar to ZmAV1 across 282 amino acids and 98.58% identical across 846 nucleotides. ZmAV2-KG consists of three contigs that are 378, 427, and 804 nt long. All three contigs mapped to unique regions of the ZmAV2 genome (Fig 1B) and are greater than 90% identical to ZmAV2 at the nucleotide level. A complete RdRP domain was found in the 804 nt contig which allowed us to estimate phylogeny and comply with the ICTV standards for species demarcation (https://ictv.global). At 93.23% nucleotide identity, ZmAV2-KG falls short of the 25% RdRP divergence criterion to be considered a new species. Consistent with these results, we found high phylogenetic branch support for the relationship between ZmAV1/ZmAV1-SD and ZmAV2/ZmAV2-KG (Fig 1A).

The amalgaviruses are known to be persistent viruses, which reside indefinitely within their host, transmit efficiently by seed, and do not generally cause outward disease symptoms [21, 54]. Amalgaviruses maintain low titers that mitigate host defense systems [55, 56], and with no recognized movement protein, amalgavirus particles likely spread during mitotic division in the same way as other persistent viruses [54, 57]. The amalgaviruses are also known to have a high degree of genetic homogeneity within species, which is consistent with our findings. Genotypes of southern tomato virus and blueberry latent virus, for example, can share ~ 99% of their respective genomes within species across a wide geographic area [50, 52]. The geographic similarity between known ZmAV genotypes and the apparent lack of disease in their hosts suggests that seagrass amalgaviruses are ecologically similar to their terrestrial counterparts, both in transmission and effect on their host.

### Endornaviridae

**Fig 2:**
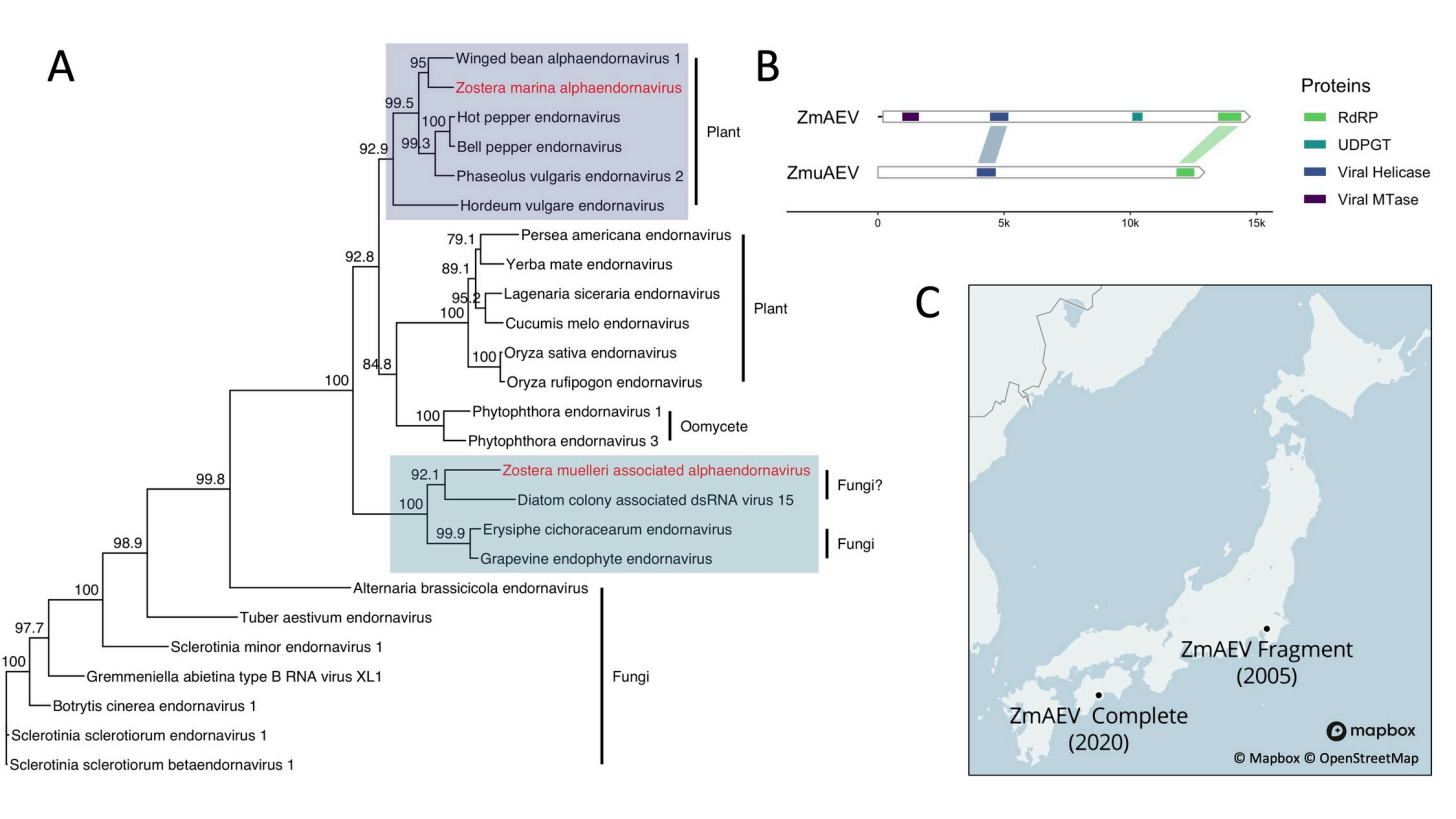
Phylogeny and genome maps for new alphaendornavirus genomes. (A) Phylogenetic placement of Zostera marina alphaendornavirus (ZmAEV) and Zostera muelleri associated alphaendornavirus (ZmuAEV) using a maximum-likelihood inference. This phylogeny was constructed with an amino acid alignment of RdRP domains. Numeric values indicate the degree of SH-like branch support (scale 0–100). Fungi/oomycete/plant labels signify the host type. (B) Genome maps for ZmAEV and ZmuAEV. White arrows represent a single ORF for both genomes. Colored segments indicate protein coding regions. Scale shows the length in nucleotides. (C) Field sites in Japan where the partial RdRP fragment [23] and complete genome (this study) for ZmAEV originated. Each label is accompanied by the year that *Z*. *marina* was sampled. This map was printed from under a CC BY license with permission from Mapbox.

We discovered two alphaendornavirus genomes associated with *Z*. *marina* and *Z*. *muelleri*. The *Endornaviridae* are persistent, capsidless [58], positive-sense ssRNA viruses with two recognized genera–*Alphaendornavirus* and *Betaendornavirus* [59]. The latter is shorter (< 10.7 kb) and is known to infect ascomycete fungi, whereas the former is longer (> 11.9 kb) and is known to infect plants, fungi, and oomycetes [59]. The genomes that we discovered belong to the *Alphaendornavirus* genus. Both are novel to this study and geographically separate from each other. We refer to these genomes as Zostera marina alphaendornavirus (ZmAEV) and Zostera muelleri associated alphaendornavirus (ZmuAEV). We found ZmAEV in the same libraries as ZmAV2-KG (collected in Japan) and assembled ZmuAEV from leaf tissue from Whangārei Harbor, New Zealand. Both genomes are complete, or near complete, with respective lengths of 14,767 and 12,947 nucleotides. ZmAEV and ZmuAEV have similar genetic architectures but differ in two respects. While each encodes a single ORF with helicase and RdRP domains from the 5´ to 3´ direction, ZmAEV has viral methyltransferase (MTase) and UDP-glucosyltransferase domains that are absent in ZmuAEV (Fig 2B). The genomic and geographic dissimilarity between ZmAEV and ZmuAEV indicates these viruses are not contaminants. Their finding, however, is surprising, given our methodology and their capsidless nature. Residual RNA*later*^TM^ that was used to preserve the original tissues may explain why these viruses did not degrade.

Our phylogenetic analysis shows that ZmAEV and ZmuAEV are distinguished by host-defined clades of known viruses (plant and fungal, respectively; Fig 2A), an observation that led us to append the designation of ZmuAEV with ‘associated,’ signifying its ambiguous origin. Regarding ZmAEV, our analysis supports the conclusion that it originates from *Z*. *marina* tissues and not co-extracted epibionts. Its closest relative is winged bean alphaendornavirus 1, and both cluster in a larger clade of plant endornaviruses. ZmuAEV, by contrast, is phylogenetically more similar to fungal viruses. Its closest relative is a diatom-associated virus, and both share a common ancestor with Erysiphe cichoracearum endornavirus and grapevine endophyte endornavirus. This leaves the possibility that ZmuAEV could derive from an epibiont; however, a definitive answer is outside the purview of this study. Roossinck *et al*. [60] discuss the modular nature of endornavirus evolution, highlighting the convergence of different source-derived domains and propose that fungi could be a vector for horizontal transmission to plants. In the rice sheath blight fungus *Rhizoctonia solani*, the horizontal transmission of an endornavirus between fungi has been established [61].

We found a partial RdRP sequence in the DNA Data Bank of Japan (accession number AB185249) that mapped to ZmAEV with 98.5% pairwise similarity. The sequence came from a study of multiple plant species that included *Z*. *marina* in Tokyo Bay (Fig 2C) [23]. The similarity between ZmAEV and the partial sequence suggests that the partial sequence may belong to the same virus. The time elapsed between sampling in the Fukuhara *et al*. [23] study and ours is approximately 15 years, which suggests that ZmAEV is relatively stable in Japanese *Z*. *marina* populations. This is consistent with the consensus view that endornaviruses are mostly non-pathogenic. While there is evidence that endornavirus infections can elicit physiological changes at the cellular level that resemble acute infections [62], the only observed phenotype with deleterious effects is cytoplasmic male sterility in *Vicia faba* [63]. Furthermore, effects, if any, may be host dependent, including potential benefits. For example, seeds of black turtle soup that were coinfected with Phaseolus vulgaris endornavirus 1 and 2 germinated faster than non-infected seeds, giving virus positive cultivars a potential selective advantage [64]. However, in a similar study in bell peppers, endornavirus positive lines were less likely to germinate than negative lines [65].

### Betaflexiviridae

**Fig 3:**
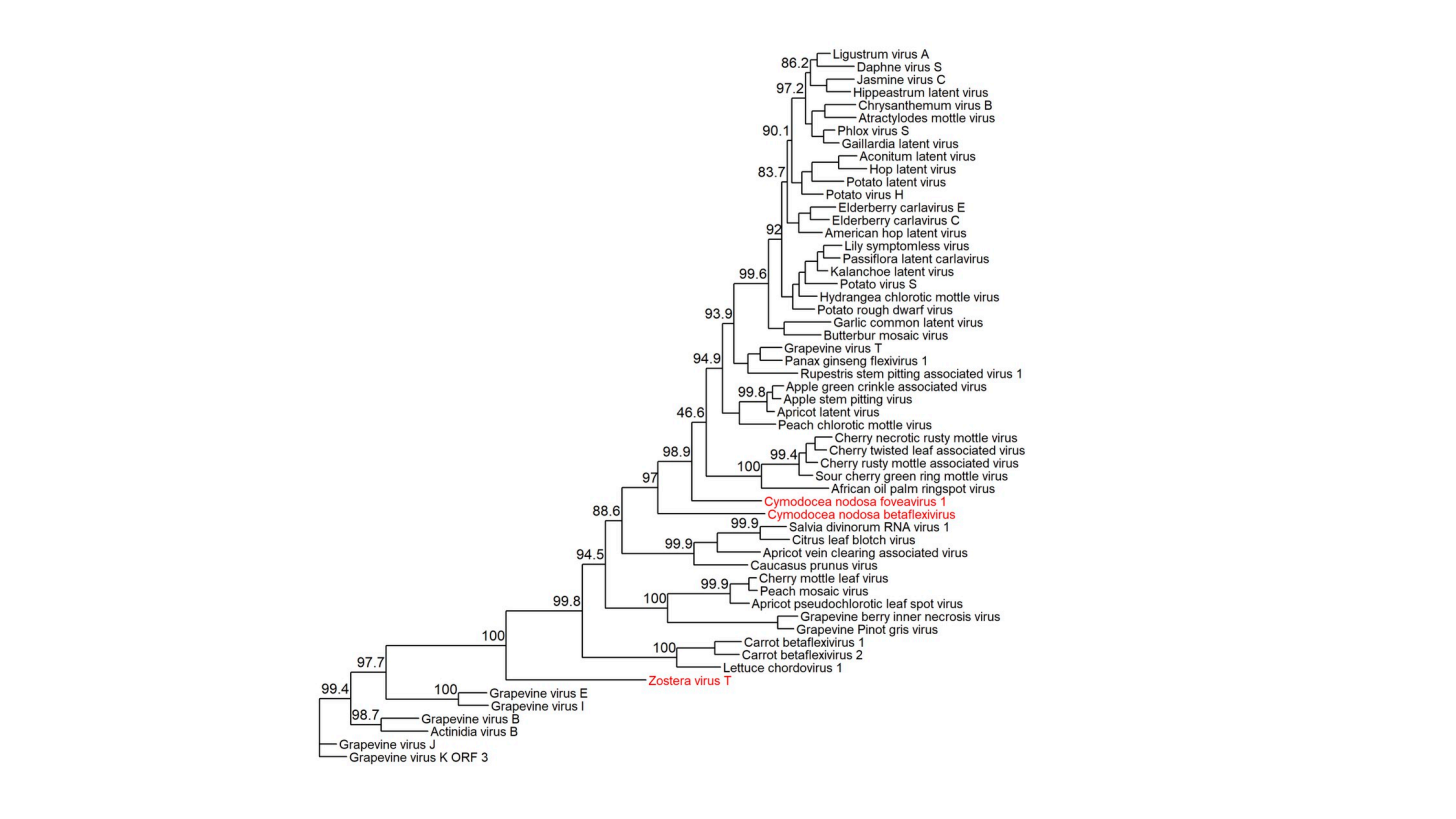
*Betaflexiviridae* phylogeny. Maximum-likelihood tree of the family *Betaflexiviridae* based on RdRP amino acids. Numeric values indicate the degree of SH-like branch support (scale 0–100). Known seagrass viruses are colored red.

We discovered two contigs in publicly available transcriptomes that belong to a virus in the *Betaflexiviridae*. This family has viruses with linear, positive-sense ssRNA genomes and filamentous, non-enveloped virions [66]. The contigs that we found came from the seagrass *C*. *nodosa* in Cadiz Bay, Spain, and are 5,347 (contig 1) and 2,847 (contig 2) nucleotides long. A phylogenetic analysis of the RdRP (contig 1) and movement protein (contig 2) strongly suggests that both contigs come from the same virus (S1 Fig), which we refer to as Cymodocea nodosa betaflexivirus (CNBV). S2 Fig shows that CNBV has three ORFs for replication, movement, and coat formation. ORF 1 encodes viral MTase, endopeptidase, viral helicase, and RdRP domains, while ORFs 2 and 3 encode the movement and coat proteins, respectively. Phylogenetically, CNBV does not fall into a clear genus. Based on pairwise distances, CNBV is most closely related to members of the *Prunevirus*, *Citrivirus*, and *Foveavirus* genera (Fig 3). Organizationally, CNBV most resembles members of the *Citrivirus* genus (S2 Fig).

As of this paper, families in the order *Tymovirales* have the greatest number of viruses associated with seagrass. Goh *et al*. [25] discovered three contigs from the genus *Tepovirus* (*Betaflexiviridae*) by mining *Z*. *muelleri* transcriptomes from Sydney, Australia. Van Bogaert *et al*. [26] discovered a potexvirus (*Alphaflexiviridae*) in *Thalassia testudinum* (turtlegrass) from Tampa Bay, Florida, USA. More recently, Bejerman and Debat [27] discovered an unclassified alphaflexivirus and foveavirus (*Betaflexiviridae*) in *C*. *nodosa* through an extensive mining project. Of those in the *Betaflexiviridae*, CNBV and the tepovirus Zostera virus T (ZVT) both have movement proteins from the 30K superfamily but differ with respect to their coat proteins. CNBV’s coat protein belongs to the flexivirus protein family (Pfam: Flexi_CP, PF00286) while ZVT’s belongs to the trichovirus family (Pfam: Tricho_coat, PF05892). CNBV’s combination of movement and Flexi_CP domains are atypical. To the best of our knowledge, only two viruses have a similar configuration, citrus leaf blotch virus and Salvia divinorum RNA virus 1 (S2 Fig), both of which belong to the *Citrivirus* genus. Most viruses with the Flexi_CP domain have triple gene block proteins for cell-to-cell movement. The architecture of the foveavirus Cymodocea nodosa foveavirus 1 is consistent with this convention (S2 Fig).

### Bromoviridae

**Fig 4:**
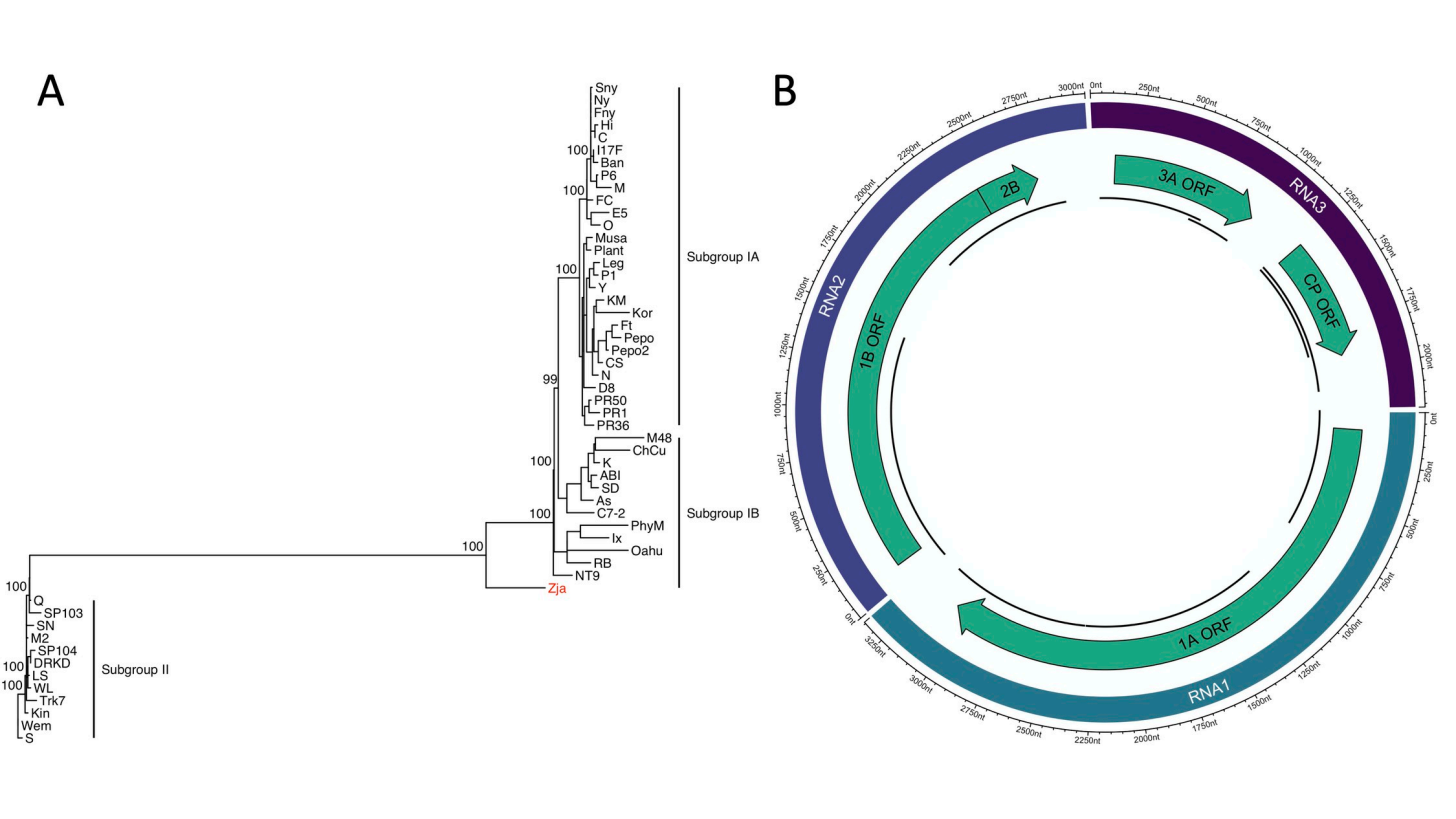
Phylogeny and contig alignments for CMV-Zja. (A) Maximum-likelihood tree of CMV strains. This tree shows the phylogenetic placement of CMV-Zja using a nucleotide alignment of the coat protein, which is the standard for differentiating subgroups. Numeric values indicate the degree of SH-like branch support (scale 0–100). CMV-Zja belongs to the 1B subgroup and is colored red. (B) Circlize plot of CMV-Zja contigs mapped to the CMV-Fny strain. Track 1 represents the complete length of RNA 1, 2, and 3. Scale markers outside of track 1 are in nucleotides. Track 2 shows the relative position of each ORF. Track 3 represents CMV-Zja contigs and their genomic position relative to CMV-Fny.

We assembled nine cucumber mosaic virus (CMV) contigs from publicly available *Z*. *japonica* transcriptomes derived from Pearl Bay, China [30]. CMV is a globally distributed agricultural pathogen [67, 68]. Furthermore, CMV has an extremely wide host range. As of Yun *et al*. [69], CMV has been documented in 1071 plant species across 521 genera and 100 families. Structurally, CMV strains have tripartite, positive-sense ssRNA genomes, and non-enveloped, icosahedral capsids [70]. The 1A and 2A ORFs on RNA 1 and 2 both participate in replication [71], and ORF 2B is involved in RNA silencing [72]. RNA 3 has two ORFs. The first ORF, 3A, encodes a movement protein, whereas the second, CP, encodes a coat protein. The contigs that we assembled map to all CMV ORFs with complete or partial coverage (Fig 4B). Their respective lengths are 296, 662, 670, 766, 863, 894, 900, 1148, and 1484 nucleotides, and they range between 83 and 91% similar to the CMV-Fny strain from the NCBI RefSeq database. A partial alignment of 182 amino acids from the RdRP domain shows that the *Z*. *japonica* strain, CMV-Zja, and CMV-Fny are 95.12% similar.

CMV strains are divided into three subgroups based on serological factors and the phylogeny of their coat protein and RNA 3 5´ untranslated region (UTR) [73]. Subgroups IA and IB are phylogenetically very similar. Subgroup II, however, is more distantly related [73]. We determined that CMV-Zja belongs to the IB subgroup using a nucleotide alignment of its coat protein with 53 other CMV strains to create a phylogenetic tree (Fig 4A). This result is consistent with the observation that most IB strains originate from Asia [74]. Our assembly did not include the 5´ UTR on RNA 3, which means we were unable to use its phylogeny.

CMV-Zja is notable because CMV strains are frequently aphid vectored. Over 80 aphid species are known to play a role in CMV transmission, and this relationship can be highly tuned between the strain of CMV and aphid species [75]. Based solely on its genome, we cannot validate how CMV-Zja was transmitted. Additionally, it is hard to assess whether CMV-Zja was introduced through contamination because its genome derives from public transcriptomes. One possibility is that CMV-Zja was transmitted to *Z*. *japonica* by an aphid vector during low tide. CMV-Zja derives from *Z*. *japonica* plants that grow in the intertidal zone, which was confirmed through personal correspondence with the lead author of Chen *et al*. [30]. Furthermore, aphids can travel long distances and inoculate new hosts rapidly [76, 77], which could provide CMV-Zja an opportunity to infect *Z*. *japonica* during low tide. However, this requires further investigation. Whether CMV can propagate continuously between *Z*. *japonica* plants (e.g., through seed transmission [68]) or whether *Z*. *japonica* becomes a dead-end host is unknown.

### Virgaviridae

**Fig 5:**
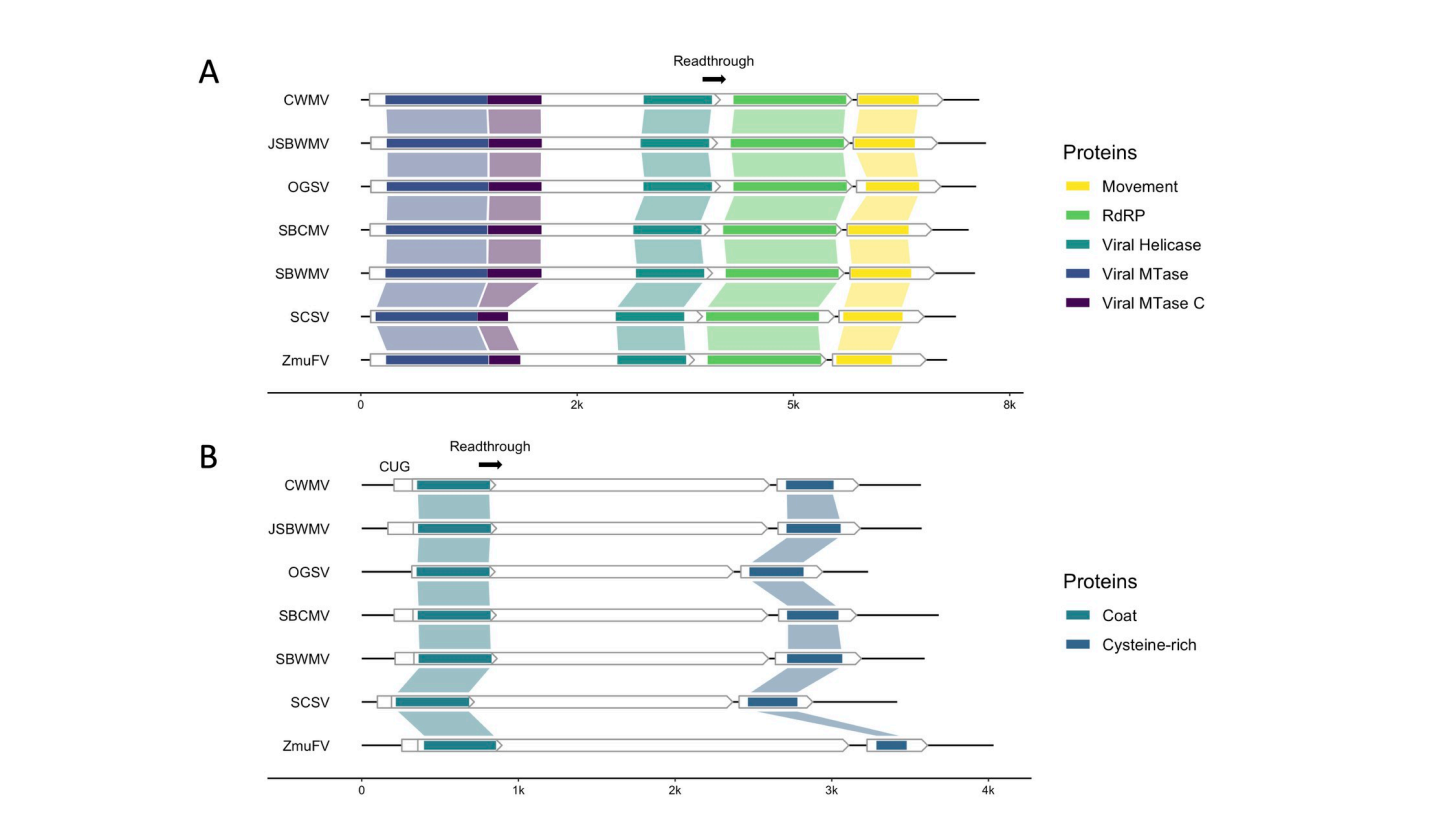
Furovirus genome maps for RNA 1 and 2. (A) RNA 1. (B) RNA 2. White arrows represent ORFs. Colored segments indicate protein coding regions. Leaky stop codons, which enable the readthrough of ORF 1 for both RNAs, can be found after the viral helicase (RNA 1) and major coat (RNA 2) domains. For all furoviruses except OGSV, a minor coat protein is predicted to be translated on RNA 2 from the non-canonical start codon CUG. Virus names/abbreviations are as follows–Chinese wheat mosaic virus (CWMV), Japanese soil-borne wheat mosaic virus (JSBWMV), oat golden stripe virus (OGSV), soil-borne cereal mosaic virus (SBCMV), soil-borne wheat mosaic virus (SBWMV), sorghum chlorotic spot virus (SCSV), Zostera muelleri furovirus (ZmuFV).

We discovered a novel furovirus in a survey of multiple *Z*. *muelleri* beds in Whangārei Harbor, New Zealand (S3 Fig). Furoviruses have bipartite genomes with linear, positive-sense ssRNA and non-enveloped, rod shaped virions with helical symmetry [78, 79]. The contigs that we assembled represent a nearly complete genome, comprise both RNAs, and contain all universally conserved proteins in the *Furovirus* genus (Fig 5). Two sites, Munro Bay and One Tree Point, contained reads that successfully mapped to the entire consensus genome. We are calling this virus Zostera muelleri furovirus, or ZmuFV, which, to the best of our knowledge, is the first instance of a furovirus naturally occurring outside of cereal grasses. Members of the *Furovirus* genus are limited, comprising only six species (not including ZmuFV), but known isolates are highly pathogenic and globally distributed.

The ZmuFV RNA 1 contig is 6,608 nucleotides long and contains three ORFs. At the 5´ end of ORF 1, ZmuFV encodes a viral MTase and its associated C terminal domain. Further downstream, ORF 1 encodes a viral helicase and leaky stop codon that enables the readthrough translation of an RdRP on ORF 2 [80]. ORF 3 encodes a 30K-like movement protein that facilitates cell-to-cell movement post infection (Fig 5A). The RNA 2 contig is 4038 nucleotides long and encodes three putative coat proteins, one major and two minor [81], and a 19kDa cysteine-rich silencing protein. The first minor coat protein is derived from the initiation of translation at a non-canonical CUG start codon and terminates at the stop codon following the major coat protein coding region (Fig 5B). The second is derived from the readthrough of the same stop codon and initiates at the canonical AUG start codon.

**Fig 6:**
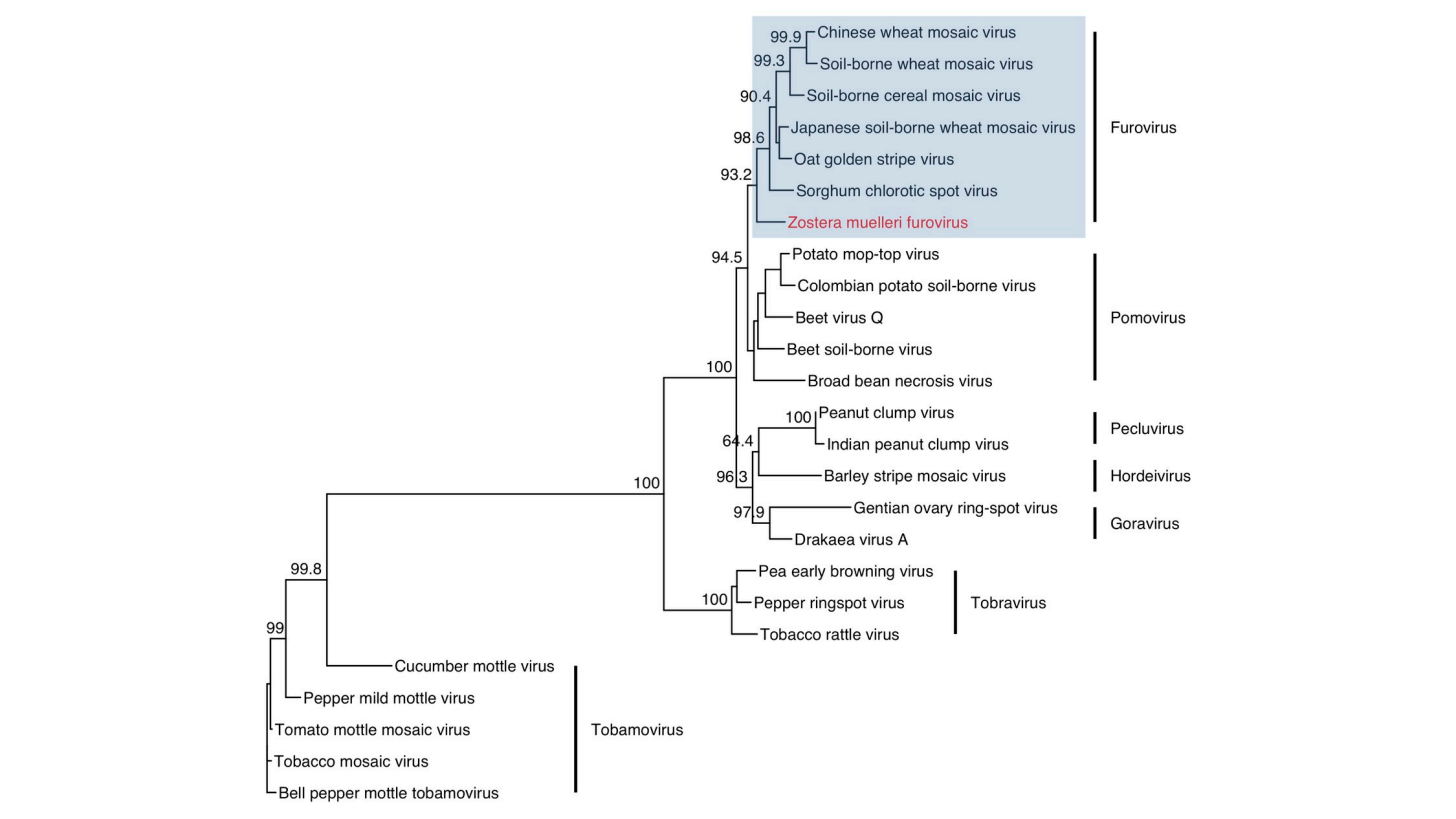
*Virgaviridae* phylogeny. Phylogenetic placement of Zostera muelleri furovirus (ZmuFV) RNA 1 using an amino acid alignment of *Virgaviridae* RdRP domains and a maximum-likelihood inference. Numeric values indicate the degree of SH-like branch support (scale 0–100).

Phylogenetically, ZmuFV diverges from the base node of the furovirus clade in the family *Virgaviridae* (Fig 6). Using RdRP derived branch length estimates, we predict that ZmuFV RNA 1 is most similar to Japanese soil-borne wheat mosaic virus and oat golden stripe virus. Our phylogenetic analysis also predicts that ZmuFV is more closely related to some members of the *Pomovirus* genus than more distantly related furoviruses, which suggests that ZmuFV may be closely related to a common ancestor of the *Furovirus* and *Pomovirus* genera. These predictions are consistent with the topology and branch length estimates from multiple coding regions (S4 Fig).

Regarding transmission, the mechanism by which ZmuFV transmits between plants remains an open question. From direct and corollary evidence [82–84], terrestrial furoviruses are believed to be vectored exclusively by *Polymyxa graminis*, a soil dwelling protist and obligate biotroph that infects root tissue [85]. Virus-vector interactions are highly specific and strongly conserved, so we speculate that ZmuFV may be vectored by a phylogenetically similar organism. While a number of plasmodiophorids have been characterized in terrestrial and freshwater systems, relatively few are known in the marine environment [86]. This is likely due to sampling bias; however, representatives associated with key marine constituents, including seagrass, have been documented [87, 88].

One line of evidence supports the potential transmission of ZmuFV by a plasmodiophorid vector. Plasmodiophorid vectored viruses almost all encode transmembrane proteins in their coat readthrough domains that enable viral translocation across membrane barriers [89]. This is supported by experimental and observational evidence [78, 90–92]. With the exception of oat golden stripe virus (OGSV), furovirus species have two predicted transmembrane domains, TM1 and TM2, as shown by Adams *et al*. [89]. However, evidence exists for a vestigial domain in OGSV (S5 Fig). Our analysis of ZmuFV predicts that two transmembrane proteins are conserved in its coat readthrough domain (S5 Fig), which is consistent with transmission by a plasmodiophorid. However, the mode of transmission for ZmuFV is unknown; and, importantly, transmission dynamics remains a significant knowledge gap in seagrass virology.

## Conclusions

The ecological and economic significance of seagrasses have inspired a great deal of interest in their microbiomes. Relatively little is known, however, about the seagrass virome, despite the important roles of viruses in plant ecology. In this study, we broadly surveyed RNA viruses in different seagrass species and uncovered a high degree of viral diversity, doubling the number of known seagrass viruses. In total, we recovered seven partial and complete RNA viruses across the species *Z*. *marina*, *Z*. *muelleri*, *Z*. *japonica*, and *C*. *nodosa*. There is little known, however, about the effects of seagrass viruses on their hosts and in the greater environment. We predict that viruses play important roles in seagrass ecology and encourage further research that explores questions related to their ecological impacts, geographic distributions, and transmission pathways.

## Supporting information

S1 Table The mean sequencing depth for all reported contigs. Contigs are listed in order of their relative positions in a 5´ – 3´ configuration.
(TIF)

S1 Fig *Betaflexiviridae* co-phylogeny. Maximum-likelihood co-phylogenetic tree comparing amino acid alignments from the RdRP (left; contig 1) and movement (right; contig 2) domains. Numeric values indicate the degree of SH-like branch support (scale 1–100).
(TIF)

S2 Fig Genome maps for a subset of viruses in the family *Betaflexiviridae*. White arrows represent ORFs. Colored segments indicate protein coding regions. Known seagrass viruses are colored red. For simplicity, because CNVB was recovered as two contigs, these contigs were concatenated by a 10-nucleotide linker between the first and second ORF.
(TIF)

S3 Fig Field sites where *Z*. *muelleri* was collected in New Zealand. ZmuFV reads mapped to our consensus genome from two out of six sites, Munro Bay and One Tree Point.
(TIF)

S4 Fig Phylogeny for six universally conserved proteins in the *Furovirus* genus. Each maximum-likelihood tree was constructed using amino acid alignments. Viral helicase, RdRP, viral MTase, and movement domains are found on RNA 1. The coat and cysteine-rich domains are found on RNA 2. Viral helicase, RdRP, and coat phylogenies include members of the *Pomovirus* genus. Branch highlights show congruency between protein coding regions within each RNA. Virus names/abbreviations are as follows–*Furovirus*–Chinese wheat mosaic virus (CWMV), Japanese soil-borne wheat mosaic virus (JSBWMV), oat golden stripe virus (OGSV), soil-borne cereal mosaic virus (SBCMV), soil-borne wheat mosaic virus (SBWMV), sorghum chlorotic spot virus (SCSV), Zostera muelleri furovirus (ZmuFV)–*Pomovirus*–beet soil-borne virus (BSBV), beet virus Q (BVQ), broad bean necrosis virus (BBNV), Columbian potato soil-borne virus (CPSBV), potato mop-top virus (PMTV).
(TIF)

S5 Fig Position and MSA of furovirus transmembrane domains. (A) Genome map of the furovirus ORF 1 readthrough domain on RNA 2. White arrows represent ORFs. Colored segments indicate protein coding regions. TM 1 (left) and 2 (right) denote transmembrane proteins. (B) Amino acid alignments of TM1 (top) and TM2 (bottom). Alignments include transmembrane and flanking regions. Arrows approximate transmembrane boundaries. Blue colors signify hydrophilic residues, while red colors signify hydrophobic residues. Purple residues indicate neutral charges. Virus names/abbreviations are as follows–Chinese wheat mosaic virus (CWMV), Japanese soil-borne wheat mosaic virus (JSBWMV), oat golden stripe virus (OGSV), soil-borne cereal mosaic virus (SBCMV), soil-borne wheat mosaic virus (SBWMV), sorghum chlorotic spot virus (SCSV), Zostera muelleri furovirus (ZmuFV).
(TIF)
