# Supplementary figures and images for: Diverse RNA viruses discovered in multiple seagrass species
Source: PLoS One. 2024 Aug 28;19(8):e0302314. doi: 10.1371/journal.pone.0302314 (PMC11356395; doi:10.1371/journal.pone.0302314)

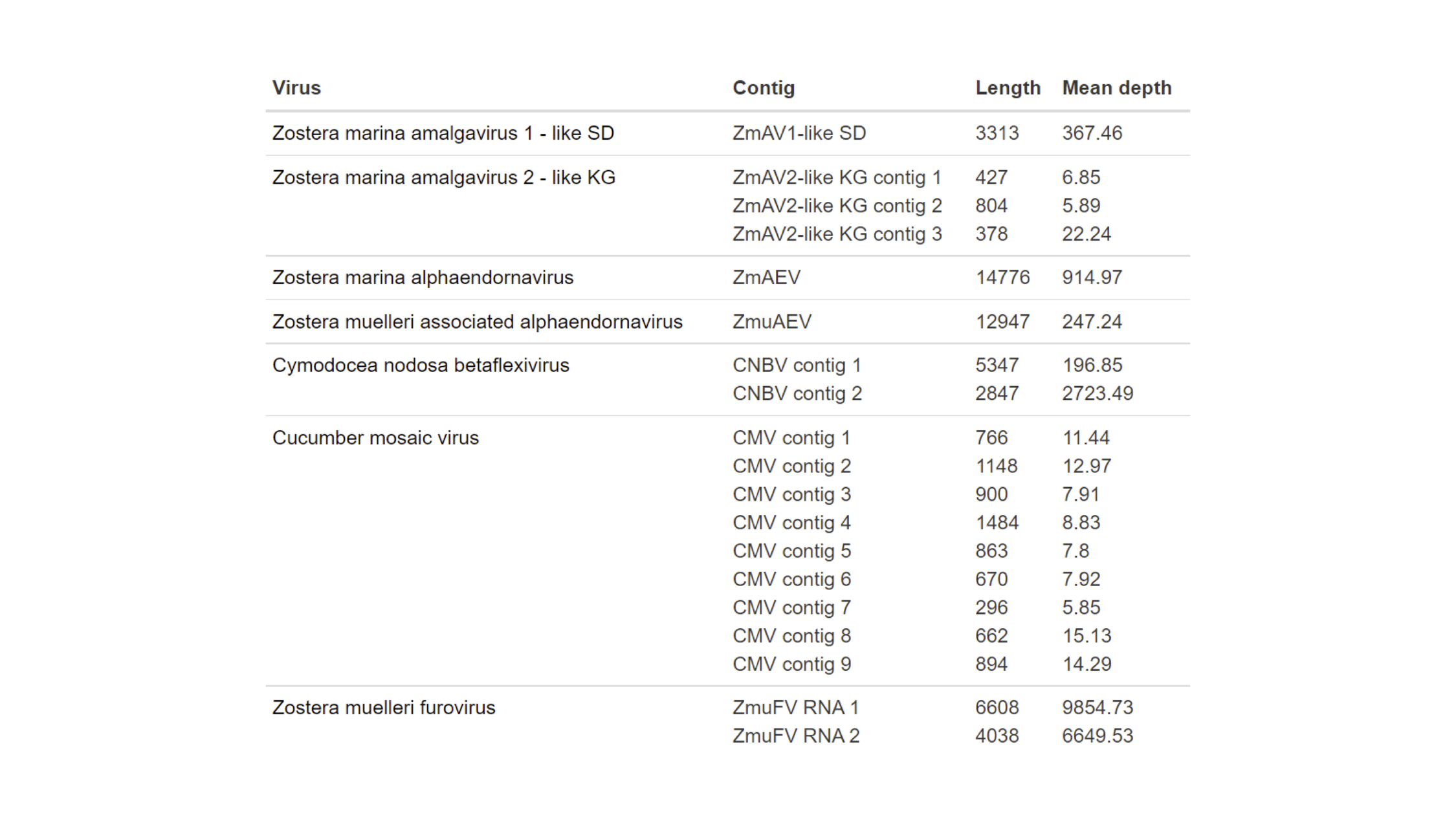

Supplement: S1 Table — Contigs are listed in order of their relative positions in a 5´ – 3´ configuration. (TIF) [file pone.0302314.s001.tif]

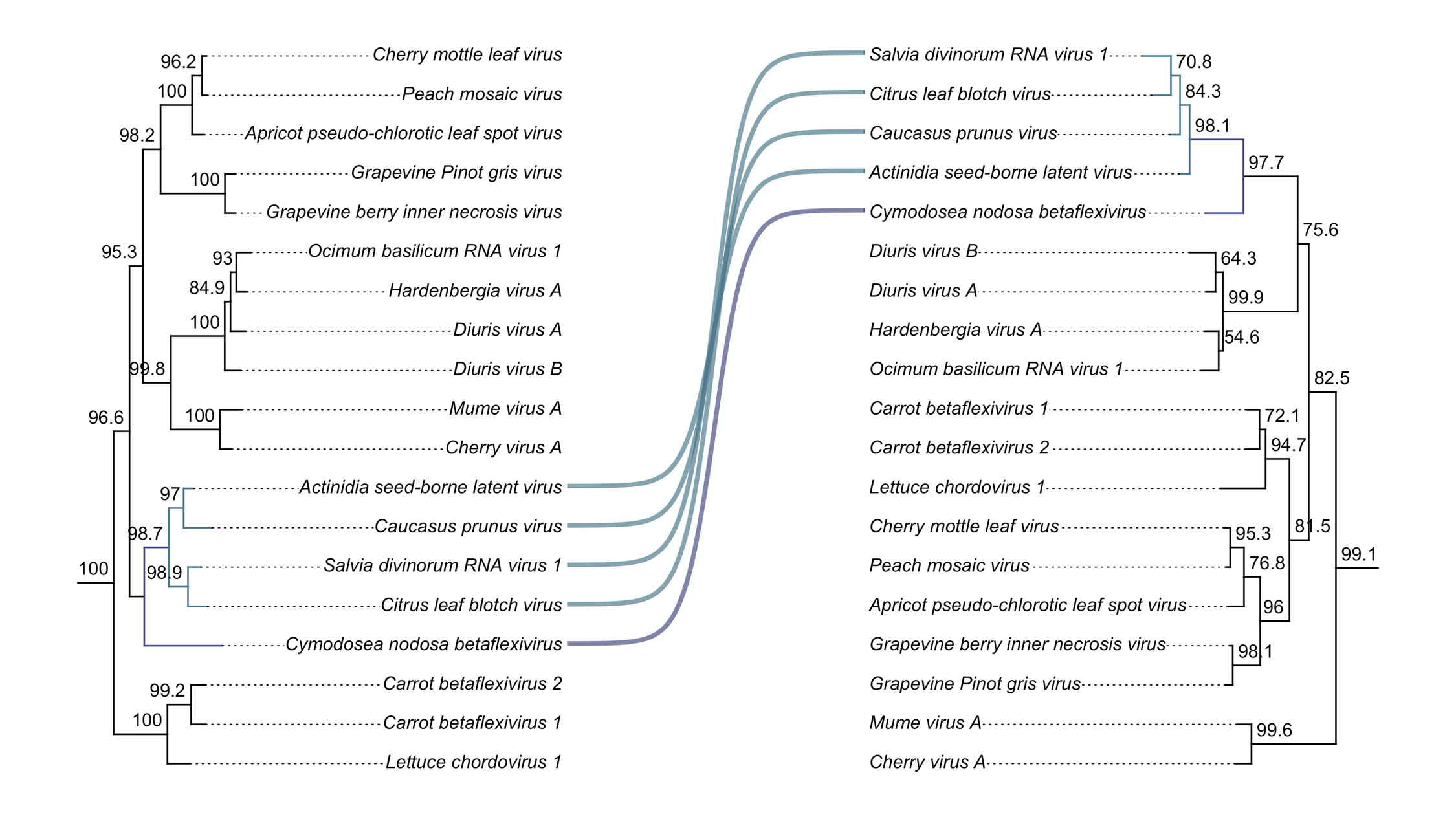

Supplement: S1 Fig — Maximum-likelihood co-phylogenetic tree comparing amino acid alignments from the RdRP (left; contig 1) and movement (right; contig 2) domains. Numeric values indicate the degree of SH-like branch support (scale 1–100). (TIF) [file pone.0302314.s002.tif]

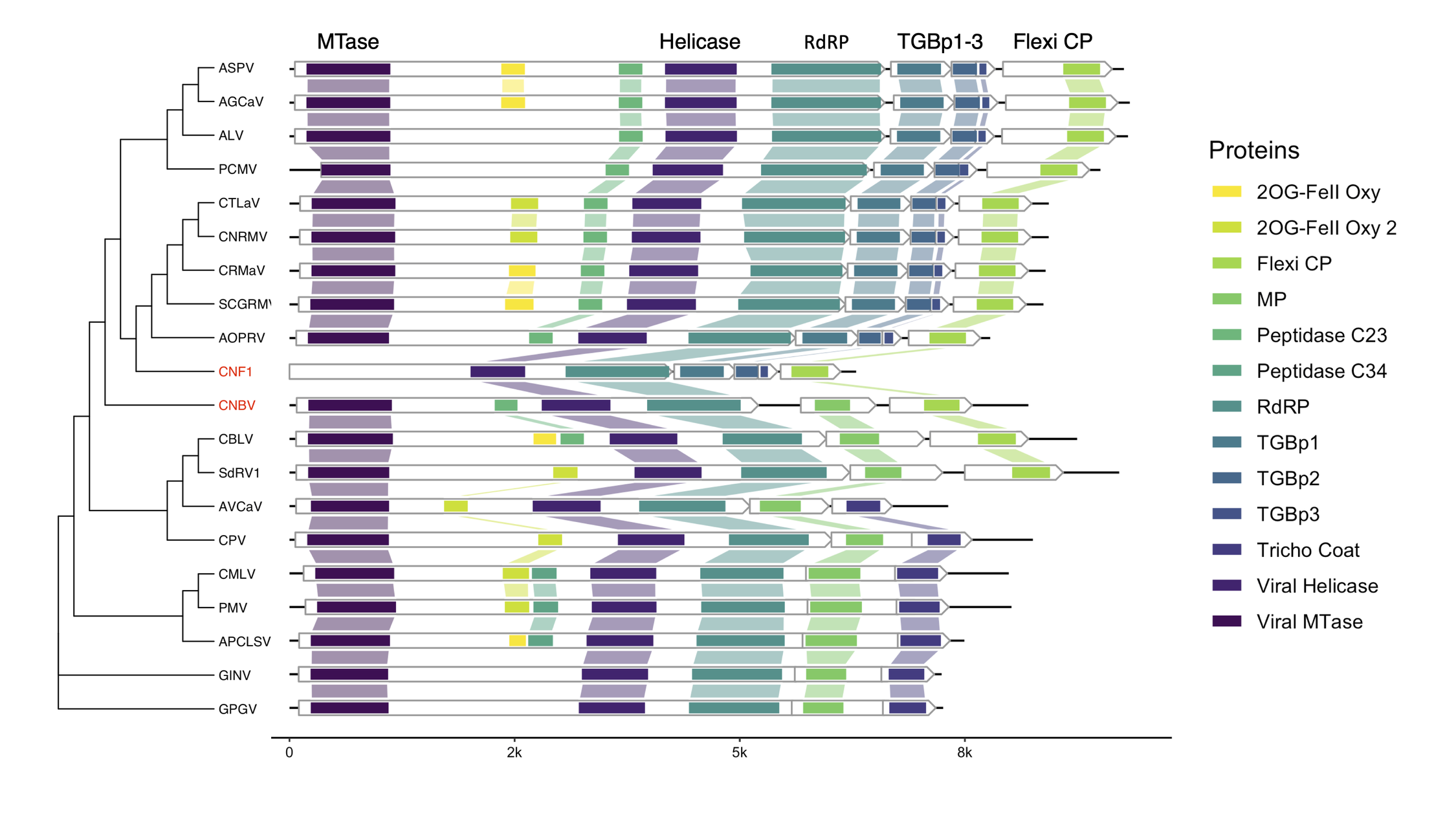

Supplement: S2 Fig — White arrows represent ORFs. Colored segments indicate protein coding regions. Known seagrass viruses are colored red. For simplicity, because CNVB was recovered as two contigs, these contigs were concatenated by a 10-nucleotide linker between the first and second ORF. (TIF) [file pone.0302314.s003.tif]

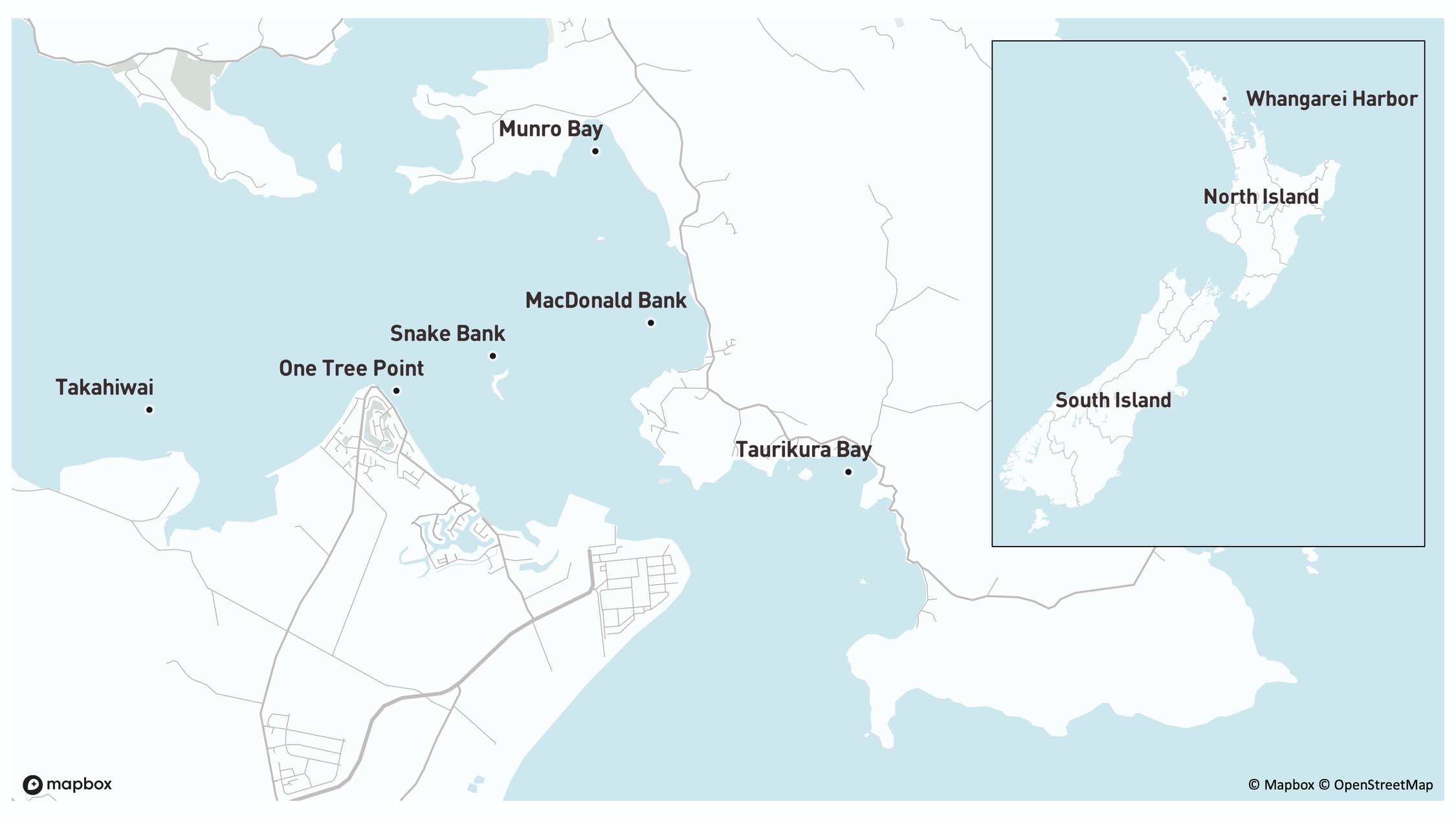

Supplement: S3 Fig — ZmuFV reads mapped to our consensus genome from two out of six sites, Munro Bay and One Tree Point. (TIF) [file pone.0302314.s004.tif]

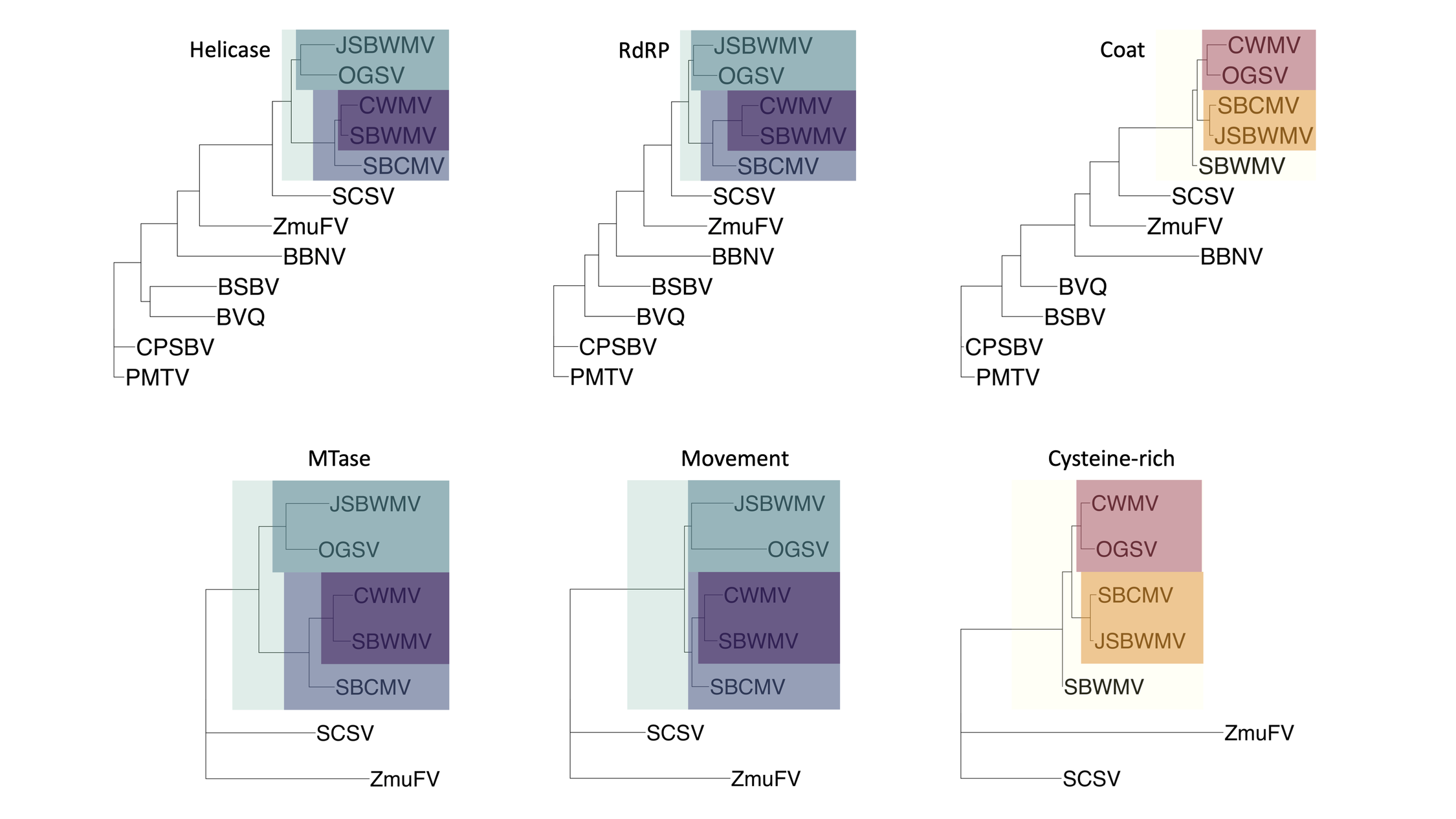

Supplement: S4 Fig — Each maximum-likelihood tree was constructed using amino acid alignments. Viral helicase, RdRP, viral MTase, and movement domains are found on RNA 1. The coat and cysteine-rich domains are found on RNA 2. Viral helicase, RdRP, and coat phylogenies include members of the Pomovirus genus. Branch highlights show congruency between protein coding regions within each RNA. Virus names/abbreviations are as follows–Furovirus–Chinese wheat mosaic virus (CWMV), Japanese soil-borne wheat mosaic virus (JSBWMV), oat golden stripe virus (OGSV), soil-borne cereal mosaic virus (SBCMV), soil-borne wheat mosaic virus (SBWMV), sorghum chlorotic spot virus (SCSV), Zostera muelleri furovirus (ZmuFV)–Pomovirus–beet soil-borne virus (BSBV), beet virus Q (BVQ), broad bean necrosis virus (BBNV), Columbian potato soil-borne virus (CPSBV), potato mop-top virus (PMTV). (TIF) [file pone.0302314.s005.tif]

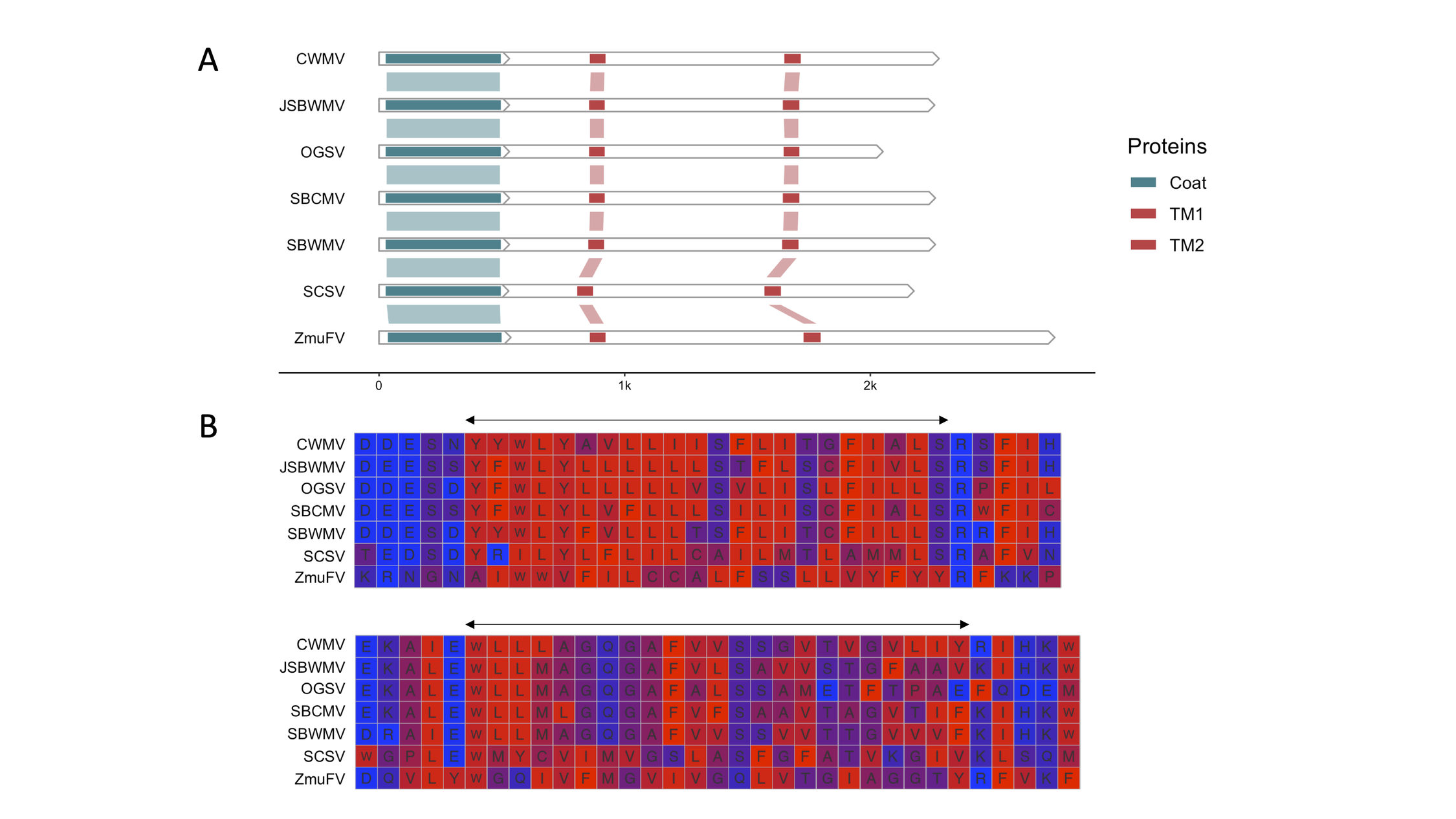

Supplement: S5 Fig — (A) Genome map of the furovirus ORF 1 readthrough domain on RNA 2. White arrows represent ORFs. Colored segments indicate protein coding regions. TM 1 (left) and 2 (right) denote transmembrane proteins. (B) Amino acid alignments of TM1 (top) and TM2 (bottom). Alignments include transmembrane and flanking regions. Arrows approximate transmembrane boundaries. Blue colors signify hydrophilic residues, while red colors signify hydrophobic residues. Purple residues indicate neutral charges. Virus names/abbreviations are as follows–Chinese wheat mosaic virus (CWMV), Japanese soil-borne wheat mosaic virus (JSBWMV), oat golden stripe virus (OGSV), soil-borne cereal mosaic virus (SBCMV), soil-borne wheat mosaic virus (SBWMV), sorghum chlorotic spot virus (SCSV), Zostera muelleri furovirus (ZmuFV). (TIF) [file pone.0302314.s006.tif]
